# Bacterial Diversity and Bioprospecting for Cold-Active Hydrolytic Enzymes from Culturable Bacteria Associated with Sediment from Nella Fjord, Eastern Antarctica

**DOI:** 10.3390/md9020184

**Published:** 2011-01-31

**Authors:** Yong Yu, Hui-Rong Li, Yin-Xin Zeng, Bo Chen

**Affiliations:** SOA Key Laboratory for Polar Science, Polar Research Institute of China, Shanghai, 200136, China; Email: lihuirong@pric.gov.cn (H.-R.L.); zengyinxin@pric.gov.cn (Y.-X.Z.); chenbo@pric.gov.cn (B.C.)

**Keywords:** psychrophilic, heterotrophic bacteria, diversity, cold-active enzymes, Antarctica

## Abstract

The diversity and cold-active hydrolytic enzymes of culturable bacteria associated with sandy sediment from Nella Fjord, Eastern Antarctica (69°22′6″ S, 76°21′45″ E) was investigated. A total of 33 aerobic heterotrophic bacterial strains were isolated at 4 °C. These bacterial isolates could be sorted into 18 phylotypes based on the 16S rRNA gene sequence belonging to four phyla, namely *Alphaproteobacteria*, *Gammaproteobacteria*, *Bacteroidetes* and *Actinobacteria*. Only seven isolates were psychrophilic, 15 isolates were moderately psychrophilic, and 11 isolates were psychrotolerant. More than 72% of the isolates required sodium chloride to grow. Esterase, β-glucosidase and proteases activities at 4 °C were detected in more than 45% of the strains while approximately 21%, 15% and 12% of the strains possessed lipase, amylase and chitinase, respectively. These results indicate that a relatively high culturable bacterial diversity is present within marine sediment of Nella Fjord and it could serve as an ideal candidate region for bioprospecting.

## 1. Introduction

Nella Fjord, located off the west coast of Bruknes Peninsula, Eastern Antarctica, is a U-shaped inlet whose narrow neck is nearly totally blocked with smaller icebergs. There, the ice cover is present for no less than ten months a year. Nella Fjord has attracted the attention of scientists as a long-term ecological monitoring site for evaluating polar ecosystems during climatic change [1]. Earlier studies in this fjord have focused on the physical characteristics of sea ice [2,3], dynamics of ice algal and phytoplankton assemblages [4], and fast ice protist community [5]. In recent years, a diverse benthic community, including more than six species of algae and 205 species of invertebrates and fish, was revealed in Nella Fjord [6,7]. The benthic microbial community in Nella Fjord thus appears to be benefiting from a flux of nutrient-rich particulates, resulting in relatively active benthic biological processes. However, benthic bacteria have received little attention in Nella Fjord [8], despite having been examined in some sediments of the Antarctic continental shelf [9,10].

In this study, we present the phylogenetic analysis of 33 bacterial isolates in order to obtain a preliminary understanding of the bacterial community composition in the sediment of Nella Fjord. In addition, these microorganisms were also used for bioprospecting for cold-active hydrolytic enzymes, which have numerous potential applications in biotechnology processes [11].

## 2. Results and Discussion

### 2.1. Isolation of Cold-Adapted Bacteria from Sandy Marine Sediment from Nella Fjord

A total of 33 bacterial strains were isolated from sandy marine sediment sampled from Nella Fjord using aerobic heterotrophic conditions at 4 °C. The bacterial number in the sample was 1.3 ± 0.5 × 10^4^ cfu/g sediment. The growth temperature ranges of all strains were tested on marine 2216 agar plates. After four weeks of incubation, growth of all strains occurred at 0 °C and no growth at 42 °C. Only seven strains were psychrophilic showing no growth above 20 °C [12], and 15 strains were moderately psychrophilic showing no growth above 25 °C [10]. Furthermore, six strains could grow between 0 and 30 °C and five strains between 0 and 37 °C indicating that they were psychrotolerant [12] (Table 1). More than 72% of the isolates required sodium chloride to grow, of which about 54% of the strains had an absolute requirement of seawater for growth (Table 1). These results suggest that most of our isolates are *bona fide* marine organisms and are well adapted to the low temperature and relatively constant salinities associated with the marine sediment from Nella Fjord. 

### 2.2. Phylogenetic Diversity

**Figure 1:**
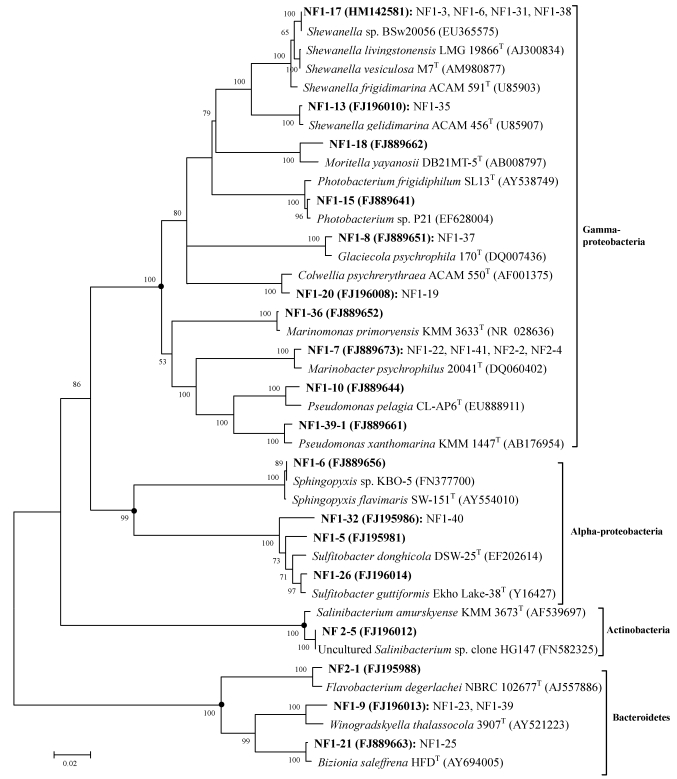
Phylogenetic relationship of the Nella Fjord isolates based on 16S rRNA gene homology. The tree was constructed using the neighbor-joining method with Kimura 2-state parameter and pairwise-deletion model analyses implemented in the program MEGA version 4.0. The resultant tree topologies were evaluated by bootstrap analysis based on 1000 replicates. Numbers at nodes represented percentage levels of bootstrap support (%). GenBank accession numbers of 16S rRNA sequences are given in parentheses. Bar, 2% sequence divergence.

Sequence similarities of our strains compared to the nearest phylogenetic neighbor ranged from 96.9 to 99.9%. Based on similarity criteria of 97% at the 16S rRNA gene, the 33 isolates sequence could be categorized into 18 phylotypes, each phylotypes representing a different taxon (Figure 1). Two strains quite likely represented novel species with less than 97% 16S rRNA gene sequence similarity [13,14]. The phylogenetic trees constructed to determine their affiliations are shown in Figure 1. 

**Table 1:** Growth temperature range, NaCl tolerance, seawater requirement and hydrolase activities of 33 bacterial isolates from sandy sediment sample from Nella Fjord.

Genus	Strain	Temperature range (°C)	NaCl tolerance (%)	Seawater requirement ^†^	Hydrolase activities *						
					Proteae	Esterase	Lipase	Chitinase	Amylase	β−Γλυχοσιδασε	β-Galactosidase
**Alphaproteobacteria**											
*Sphingopyxis*	NF1-6	0-20	1-6	+	−	+	−	−	−	−	−
*Sulfitobacter*	NF1-5	0-25	1-5	+	−	+	+	−	−	−	−
	NF1-26	0-25	1-5	+	+	+	+	−	−	−	−
	NF1-32	0-25	1-5	+	−	−	−	−	−	−	−
	NF1-40	0-25	1-5	+	−	−	−	−	−	−	−
**Gammaproteobacteria**											
*Colwellia*	NF1-19	0-25	1-5	+	−	−	+	−	+	−	−
	NF1-20	0-25	1-5	+	+	−	−	−	−	+	−
*Glaciecola*	NF1-8	0-20	1-6	−	−	+	−	−	−	−	−
	NF1-37	0-20	1-6	−	−	−	+	−	−	+	−
*Marinomona*	NF1-36	0-30	0-6	−	−	−	−	−	−	−	+
*Marinobacter*	NF1-7	0-25	2-8	−	−	+	−	−	−	+	−
	NF1-22	0-25	2-8	−	−	+	−	−	−	−	−
	NF1-41	0-25	2-8	−	−	+	−	−	−	+	−
	NF2-2	0-25	2-8	−	−	+	−	−	−	+	−
	NF2-4	0-25	2-8	−	−	+	−	−	−	+	−
*Moritella*	NF1-18	0-20	1-6	+	+	−	−	−	−	−	−
*Photobacterium*	NF1-15	0-20	1-4	+	+	−	+	−	+	+	−
*Pseudomonas*	NF1-10	0-30	0.5-8	−	−	+	+	−	+	−	−
	NF1-39-1	0-37	0-8	−	+	+	+	−	+	+	−
*Shewanella*	NF1-3	0-25	0-9	−	+	−	−	−	−	+	+
	NF1-16	0-25	0-9	−	+	+	−	−	−	+	−
	NF1-17	0-30	0-5	−	+	+	−	+	−	−	−
	NF1-13	0-20	1-6	+	−	−	−	+	−	+	−
	NF1-31	0-30	0-6	−	−	−	−	−	−	+	−
	NF1-35	0-20	1-6	+	−	−	−	+	−	−	−
	NF1-38	0-30	0-5	−	+	+	−	+	−	+	−
**Bacteroidetes**											
*Bizionia*	NF1-21	0-25	1-6	+	+	−	−	−	−	−	−
	NF1-25	0-25	1-6	+	+	−	−	−	−	−	−
	NF2-1	0-30	0-5	−	−	−	−	−	+	+	−
*Flavobacterim*	NF1-9	0-37	1-8	−	+	+	−	−	−	+	−
	NF1-23	0-37	1-8	−	+	+	−	−	−	+	+
	NF1-39	0-37	1-8	−	+	+	−	−	−	+	−
**Actinobacteria**											
*Salinibacterium*	NF2-5	0-37	0-10	−	+	−	−	−	−	−	−

^†^ No growth occurs in only the presence of Na^+^;

* All the strains were negative for cellulase and agarase activities;

+ positive, − negative.

Out of the 17 Gram-negative phylotypes, 10 belonged to the phylum *Gammaproteobacteria*, four to the phylum *Alphaproteobacteria* and three to the phylum *Bacteroidetes*. The one Gram-positive strain was affiliated to the genus *Salinibacterium* and showed 100% similarity with the uncultured *Salinibacterium* sp. clone HG147 from intestinal content and mucus of farmed seahorses (*Hippocampus guttulatus*) [15]. Further, we found that one of our psychrophilic strain (NF1-6) shared identical 16S rRNA gene sequences (1447 bp) with the Arctic psychrophilic isolate *Sphingopyxis* sp. KBO-5 from Kongsfjorden marine sediment [16], suggesting for the first time that psychrophilic bacteria are possibly dispersed between poles at the species level [17,18]. Additional work should be conducted to confirm whether these bacteria compose the same species [14,19]. 

### 2.3. Hydrolytic Enzyme Activities of the Isolates

The production of hydrolytic enzymes at 4 °C by the 33 isolates is summarized in Table 1. Isolates that are able to degrade tributyrin, esculin, skim milk, olive oil, starch, chitin and lactose accounted for, respectively, up to 51.5, 51.5, 45.5, 21.2, 15.2, 12.1 and 0.9%. No hydrolysis was observed on cellulose and agar. Esterase was produced by members of all tested genera, with the exception of *Colwellia*, *Marinomonas*, *Moritella*, *Photobacterium*, *Bizionia* and *Salinibacterium* isolates. The ability to degrade esculin was almost equally distributed among the isolates of Gammaproteobacteria and Bacteroidetes, but not Alphaproteobacteria and Actinobacteria. Skim milk was also hydrolyzed by most of our strains at 4 °C.However, *Sphingopyxis*, *Glaciecola*, *Marinomonas* and *Marinobacter* isolates did not produce proteases. Lipase activity was detected in members of *Sulfitobacter*, *Colwellia*, *Glaciecola*, *Photobacterium* and *Pseudomonas*. Chitin was exclusively hydrolyzed by the members of *Shewanella*. Amylase positive isolates were members of *Colwellia*, *Photobacterium*, *Pseudomonas* and *Bizionia*. The three β-galactosidases producers were distributed among the members of *Marinomonas*, *Shewanella* and *Flavobacterium*.

The ability of the newly isolated psychrophilic or psychrotolerant strains to produce a broad spectrum of cold-active enzymes is of great interest for both fundamental research and industrial applications. The high number of esterase-producing, β-glucosidase-producing and/or protease-producing bacteria among the isolated strains (Table 1) could be related to the natural habitat from which the microorganisms were isolated. Seabed photography revealed the presence of extensive benthic populations, including urchins, red algae, holothuriae and ascidia [6]. Most of the organic matter in the benthic communities is primarily produced in the form of high-molecular-weight compounds, which are too large for direct uptake by bacteria [20]. Before these polymeric compounds can be incorporated into microbial cells, they must first be degraded by a series of extracellular hydrolytic enzymes. This process is often the rate-limiting step of organic matter utilization by microorganisms [21]. A vast variety of cold-active biopolymer-degrading enzymes detected in this study indicate that isolated bacteria can contribute significantly to the hydrolysis of the major organic constituents (esters, proteins, α- and β-linked polysaccharides) and are, therefore, involved in carbon cycling and nitrogen cycling in the sediment of Nella Fjord. 

Diverse cold-active enzymes detected in this study may find applications in various industries (food, detergents, pharmaceutical, biofuels, *etc.*) [11]. Cold-active proteases, lipases and amylases can be used to develop detergent ingredients capable of working efficiently at low to medium temperatures. Due to their high catalytic activity under low-temperature conditions that minimize spoilage and alterations in flavor and nutritional values, cold-active enzymes are particularly attractive for the processing of foods. Cold-active proteases and lipases can serve as rennet substitutes and accelerate the maturation of slow-ripening cheeses. Cold-active proteases can also be used for tenderization and taste improvement of refrigerated meat products. Cold-active β-glucosidase can be used in the wine industry to improve the aroma of wines owing to their ability to catalyze transglycosylation reactions. In the dairy industry, cold-active β-galactosidases will reduce the lactose content of milk at low temperatures to improve the digestibility of dairy products for lactose-intolerant consumers. Cold-active β-galactosidases have also been shown to possess transglycosylation activities that can be used to produce galacto-oligosaccharides, a class of additives in probiotic food items. In addition, cold-active esterase and β-glucosidase are of considerable interest to enable cost-effective lignocellulose biomass conversion, thus facilitating the development of an economically-viable ethanol production from agricultural waste, forestry waste, energy crops, and municipal solid waste.

## 3. Experimental Section

### 3.1. Sediment Sample

The sandy marine sediment sample was collected from the Nella Fjord, Prydz Bay, Eastern Antarctica (69°22′6″ S, 76°21′45″ E) at a water depth of 20 m by Boris I. Sirenko from the Zoological Institute of Russian Academy of Sciences when he dived for investigation of the benthic fauna on 12 January 2007. The *in situ* temperature of the sample was −1.5 °C, and the *in situ* salinity was 35‰. This sediment sample was stored in sterilized plastic bags (250 mL) and transported to the laboratory at temperatures between 0 and 4 °C. 

### 3.2. Isolation of Bacterial Strains

In the laboratory, one gram of wet sediment sample was mixed with 99 mL of sterilized seawater supplemented with 10 glass beads (Diameter 2-3 mm) shaken at 4 °C for 1 h at 300 repetitions/min. The suspension was further diluted (1:10) in the sterilized seawater and spread onto three different marine agars. These included 1/10-strength marine R2A agar [22], 1/10-strength marine 2216 agar (Difco) and natural seawater (NSW) agar. The plates were incubated in the dark at 4 °C for up to 8 weeks. Colonies from various agar plates were picked on the basis of differing colony morphologies. Isolates were obtained in pure culture after three successive transfers to fresh agar medium and stored at −80 °C in marine 2216 broth (Difco) supplemented with 30% (v/v) glycerol. 

*3.3. 16* *S rDNA Amplification, Sequencing*

Total genomic DNA for 16S rDNA amplification was isolated from 1 mL of bacteria grown to late log phase in marine 2216 broth (Difco) and purified by kit according to the manufacturer’s instruction (BioDev, Beijing, China). Nearly full-length 16S rRNA gene was amplified by PCR using universal primers 8F (5′-AGA GTT TGA TCC TGG CTC AG-3′) and 1492r (5′-GGT TAC CTT GTT ACG ACT T-3′) [23]. The PCR mixtures (final volume, 50 µL) contained: 100 ng of the extracted DNA as a template, 5.0 µL of 10× PCR buffer (Sangon, Shanghai, China), each deoxynucleoside triphosphate at a concentration of 40 µM, 0.2 µM of each primer, and 1 U Taq DNA polymerase (TaKaRa, Japan). PCR amplification was performed with an Eppendorf Mastercycler Gradient (Eppendorf, Germany), and the following program was used: initial denaturation at 95 °C for 4 min, followed by 25 cycles of denaturation at 95 °C for 1 min, annealing at 50 °C for 1 min and extension at 72 °C for 2 min, with a final extension at 72 °C for 10 min. The PCR products were pooled and purified with the gel extraction kit (Watson, Shanghai, China), and ligated into the pMD 18-T vector (TaKaRa, Japan). The hybrid vectors transformed into *Escherichia coli* DH5α competent cells. Recombinants were selected using Luria-Bertani (LB) indicator plates containing 100 µg of ampicillin per mL, 80 µg of X-Gal (5-bromo-4-chloro-3-indolyl-β-d-galactopyranoside) per mL, 0.5 mM IPTG (isopropyl-β-d-thiogalactopyranoside). White clones were sequenced by an ABI PRISM 3730 sequencer at Shanghai Sangon Biological Engineering Technology & Services Co., Ltd. 

### 3.4. Phylogenetic Analysis

The sequences, 1427 nucleotides (nt) to 1515 nt, depending on the isolate, were compared with the data available in the RDPII (Ribosomal Database Project II) using the sequence match tool, to determine the relative phylogenetic positions. The identification of phylogenetic neighbors and the calculation of pairwise 16S rDNA sequence similarities were achieved using the EzTaxon server [24]. In addition, the sequences were compared to sequences within the NCBI database [25] using BLASTN. Sequences were aligned using Clustal X1.8 [26] with most closely homologous bacterial type strains’ 16S rDNA sequences retrieved from GenBank. Alignments were edited manually using BioEdit Sequence Alignment Editor version 5.0.9 [27] and regions of ambiguous alignment were removed. The phylogenetic tree was constructed using the neighbor-joining method [28] with Kimura 2-state parameter and pairwise-deletion model analyses implemented in the program MEGA version 4 [29]. The resultant tree topologies were evaluated by bootstrap analysis based on 1000 replicates. DNA sequences were deposited to GenBank under Accession numbers FJ195980, FJ195981, FJ195982, FJ195983, FJ195984, FJ195985, FJ195986, FJ195987, FJ195988, FJ195989, FJ195990, FJ196008, FJ196009, FJ196010, FJ196011, FJ196012, FJ196013, FJ196014, FJ196025, FJ196026, FJ889640, FJ889641, FJ889644, FJ889651, FJ889652, FJ889656, FJ889661, FJ889662, FJ889673, FJ889663, EU365575 and HM142581.

### 3.5. Growth Temperature Range, Sodium Chloride Tolerance and Seawater Requirement

Growth of isolates in a temperature range of 0-42 °C was studied on marine 2216 agar. The strains were cultivated at 0, 4, 10, 15, 20, 25, 30, 37 and 42 °C for 2 to 4 weeks. Growth was monitored at up to 64× magnification by using a Leica stereoscope (Leica Microscopy Systems Ltd., Switzerland). The tolerance for NaCl (0-15%; w/v) was tested in medium containing (1 000 mL deionized water) 5 g MgCl_2_, 2 g MgSO_4_, 0.5 g CaCl_2_, 1 g KCl, 5 g peptone and every concentrations of NaCl at pH 7.5, adjusted with KOH [30]. Growth requirement for seawater was tested in marine 2216 broth (Difco) and in a medium containing (1000 mL deionized water) 30 g NaCl, 5 g peptone and 1 g Yeast Extract at pH 7.5, adjusted with NaOH. Broth was inoculated with pre-culture and incubated at 15 °C with slight shaking (30 rpm) for 48 and 96 h. Growth was determined by measuring OD at 600 nm.

### 3.6. Hydrolase Activities

Protease, esterase, lipase, chitinase, amylase, cellulase, β-glucosidase and β-galactosidase were detected on diagnostic plates after incubation at 4 °C for 2 to 4 weeks. Protease activity was tested on skim milk plates containing (per liter NSW) 50 g of skim milk, 5 g of peptone and 1 g of yeast extract. Clearing zones around the colonies were used as an indication of protease activity. Esterase activity was detected on tributyrin plates containing (per liter NSW) 5 mL of tributyrin, 5 g of peptone and 1 g of yeast extract. Formation of clearing zones around colonies was used to indicate esterase activity. Lipase activity was detected by using Rhodamine B plus olive oil agar plates, as described previously [31]. Chitin hydrolysis was tested according to the procedure of West and Colwell [32]. Amylase activity was determined on starch plates containing (per liter NSW) 2 g of soluble starch, 5 g of peptone and 1 g of yeast extract. The starch hydrolysis was detected by flooding plates with Lugol’s iodine solution after 2 weeks of incubation. The β-glucosidase activity was tested on esculin plates containing (per liter NSW) 1 g of esculin, 0.5 g of ferric ammonium citrate, 5 g of peptone and 1 g of yeast extract. Black to reddish brown colors that appeared in the medium surrounding the colonies was considered to be positive [33]. The β-galactosidase was determined qualitatively by the appearance of blue-colored colonies on MA supplemented with 0.1% (w/v) lactose and 0.002% (w/v) Xgal (5-bromo-4-chloro-3-indoyl-β-d-galactopyranoside). Cellulase activities were screened on MA supplemented with 0.5% (w/v) carboxymethyl cellulose. After 2 weeks of incubation cellulose hydrolysis was determined with Congo red staining [34]. In the case of agarase activity, a positive reaction was noticed when sunken colonies occurred on MA.

## 4. Conclusions

The 33 bacterial strains categorized into 18 phylotypes indicated that a relatively high diversity was present within the bacterial community associated with sandy marine sediment from Nella Fjord, Eastern Antarctica. A vast variety of cold-active biopolymer-degrading enzymes detected in this study indicated that isolated bacteria could contribute significantly to the hydrolysis of the major organic constituents (esters, proteins, α- and β-linked polysaccharides) and are therefore involved in carbon cycling and nitrogen cycling in the sediment of Nella Fjord. Furthermore, the data obtained in this study also confirmed that Nella Fjord could serve as an ideal candidate region for bioprospecting.

## References

[1] Melnikov I.A., Leitchenkov G.L., Troshichev O.A., Melnikov I.A. (2008). Life siences. RUSSIA National Report to SCAR for Year: 2008.

[2] Tang S., Kang J., Zhou S., Li Z. (2005). Sea ice characteristics between the middle Weddell Sea and the Prydz Bay, Antarctica during the austral summer of 2003. Acta Oceanol. Sin..

[3] Tang S., Qin D., Ren J. (2007). Structure, salinity and isotopic composition of multi-year landfast sea ice in Nella Fjord, Antarctica. Cold Reg. Sci. Technol..

[4] He J., Chen B. (2000). Seasonal change of ice algal and phytoplankton assemblages in the Nella Fjord near Zhongshan Station, East Antarctica. Chin. J. Polar Sci..

[5] Thomson P.G., McMinn A., Kiessling I., Watson M., Goldworthy P.M. (2006). Composition and succession of dinoflagellates and chrysophytes in the upper fast ice of Davis Station, East Antarctica. Polar Biol..

[6] Sirenko B.I., Gagayev S.Yu., Dzhurinsky V.P. (2007). *Hydrobiological Research Activities in the Nella Fjord, Prydz Bay*; IPY-2007/08 NEWS, N 9–10 (November/December 2007).

[7] Melnikov I.A., Klepikov A., Leitchenkov G., Melnikov I.A. (2010). Life siences. RUSSIA National Report to SCAR for Year: 2009.

[8] Zhang X.Y., Zhang Y.J., Yu Y., Li H.J., Gao Z.M., Chen X.L., Chen B., Zhang Y.Z. (2010). Neptunomonas antarctica sp.nov., isolated from marine sediment. Int. J. Syst. Evol. Microbiol..

[9] Bowman J.P., McCammon S.A., Gibson J.A.E., Robertson L., Nichols P.D. (2003). Prokaryotic Metabolic Activity and Community Structure in Antarctic Continental Shelf Sediments. Appl. Environ. Microbiol..

[10] Helmke E., Weyland H. (2004). Psychrophilic *versus* psychrotolerant bacteria-occurrence and significance in polar and temperate marine habitats. Cell Mol. Biol..

[11] Huston A.L., Margesin R., Schinner F., Max J.-C., Gerday C. (2008). Biotechnological Aspects of Cold-Adapted Enzymes. Psychrophiles: From Biodiversity to Biotechnology.

[12] Morita R.Y. (1975). Psychrophilic bacteria. Bacteriol. Rev..

[13] Wayne L.G., Brenner D.J., Colwell R.R., Grimont P.A.D., Kandler O., Krichevsky M.I., Moore L.H., Moore W.E.C., Murray R.G.E., Stackebrandt E., Starr M.P., Trüper H.G. (1987). Report of the ad hoc committee on reconciliation of approaches to bacterial systematics. Int. J. Syst. Bacteriol..

[14] Stackebrandt E., Goebel B.M. (1994). Taxonomic note: a place for DNA-DNA reassociation and 16S rRNA sequence analysis in the present species definition in bacteriology. Int. J. Syst. Bacteriol..

[15] Balcazar J.L., Lee N.M., Pintado J., Planas M. (2010). Phylogenetic characterization and *in situ* detection of bacterial communities associated with seahorses (*Hippocampus guttulatus*) in captivity. Syst. Appl. Microbiol..

[16] Srinivas T.N.R., Nageswara Rao S.S.S., Vishnu Vardhan Reddy P., Pratibha M.S., Sailaja B., Kavya B., Hara Kishore K., Begum Z., Singh S.M., Shivaji S. (2009). Bacterial Diversity and Bioprospecting for Cold-Active Lipases, Amylases and Proteases, from Culturable Bacteria of Kongsfjorden and Ny-Ålesund, Svalbard, Arctic. Curr. Microbiol..

[17] Staley J.T., Gosink J.J. (1999). Poles apart: biodiversity and biogeography of sea ice bacteria. Ann. Rev. Microbiol..

[18] Zeng Y., Zheng T., Yu Y., Chen B., He J. (2010). Relationships between Arctic and Antarctic *Shewanella* strains evaluated by a polyphasic taxonomic approach. Polar Biol..

[19] Fox G.E., Wisotzkey J.D., Jurtshuk P. (1992). How close is close: 16S rRNA sequence identity may not be sufficient to guarantee species identity. Int. J. Syst. Bacteriol..

[20] Chrost R.J., Chrost R.J. (1991). Environmental control of the synthesis and activity of aquatic microbial ectoenzymes. Microbial Enzymes in Aquatic Environments.

[21] Hoppe H.G., Chrost R.J. (1991). Microbial extracellular enzyme activity: a new key parameter in aquatic ecology. Microbial Enzymes in Aquatic Environments.

[22] Suzuki M.T., Rappé M.S., Haimberger Z.W., Winfield H., Adair N., Strobel J., Giovannoni S.J. (1997). Bacterial diversity among small-subunit rRNA Gene clones and cellular isolates from the same seawater sample. Appl. Environ. Microbiol..

[23] Weisburg W.G., Burns S.M., Pelletier D.A., Lane D.J. (1991). 16S ribosomal DNA amplification for phylogenetic study. J. Bacteriol..

[24] Chun J., Lee J.H., Jung Y., Kim M., Kim S., Kim B.K., Lim Y.W. (2007). EzTaxon: a web-based tool for the identification of prokaryotes based on 16S ribosomal RNA gene sequences. Int. J. Syst. Evol. Microbiol..

[25] National Center for Biotechnology Information. http://www.ncbi.nlm.nih.gov/.

[26] Thompson J.D., Gibson T.J., Plewniak F., Jeanmougin F., Higgins D.G. (1997). The CLUSTAL_X windows interface: flexible strategies for multiple sequence alignment aided by quality analysis tools. Nucleic Acids Res..

[27] Hall T.A. (1999). BioEdit: a user-friendly biological sequence alignment editor and analysis program for windows 95/98/NT. Nucleic Acids Symp. Ser..

[28] Saitou N., Nei M. (1987). The neighbor-joining method: a new method for reconstructing phylogenetic trees. Mol. Biol. Evol..

[29] Tamura K., Dudley J., Nei M., Kumar S. (2007). MEGA4: Molecular Evolutionary Genetics Analysis (MEGA) software version 4.0. Mol. Biol. Evol..

[30] Smibert R.M., Krieg N.R., Gerhardt P., Murray R.G.E., Wood W.A., Krieg N.R. (1994). Phenotypic characterization. Methods for General and Molecular Bacteriology.

[31] Kouker G., Jaeger K.E. (1987). Specific and sensitive plate assay for bacterial lipases. Appl. Environ. Microbiol..

[32] West P.A., Colwell R.R., Colwell R.R. (1984). Identification and classification of the *Vibrionaceae* —an overview. Vibrios in the Environment.

[33] Edberg S.C., Trepeta R.W., Kontnick C.M., Torres A.R. (1985). Measurement of active constitutive beta-d-glucosidase (esculinase) in the presence of sodium desoxycholate. J. Clin. Microbiol..

[34] Teather R.M., Wood P.J. (1982). Use of Congo red-polysaccharide interactions in enumeration and characterization of cellulolytic bacteria from bovine rumen. Appl. Environ. Microbiol..

